# Supplementary material for: Genes near tRNAs are enriched in translational machinery
Source: G3 (Bethesda). 2026 Jul 13;16(9):jkag188. doi: 10.1093/g3journal/jkag188 (PMC13535368; doi:10.1093/g3journal/jkag188)
Supplement: Supplementary file 1 [file jkag188_supplementary_data.zip › Figure_S1_G3-2026-407030.pdf]

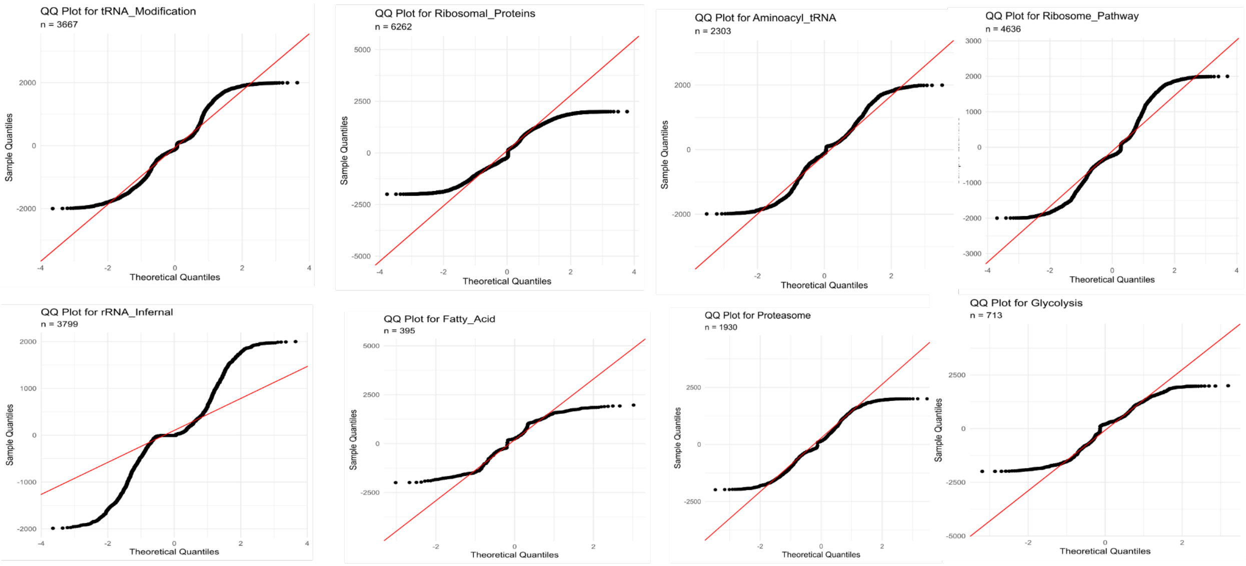

Figure S1: In order to determine which statistical test to use for tRNA modification, ribosomal protein, aminoacyl tRNA, ribosome pathway, rRNA infernal, fatty acid, glycolysis, and proteasome groups distance from tRNAs, qqplots were generated. None of the qqplots showed a normal distribution. A non-normal distribution requires a non-parametric test, so the results of the qqplots indicate the need for a Wilcoxon rank-sum test.
