# Supplementary material for: Genes near tRNAs are enriched in translational machinery
Source: G3 (Bethesda). 2026 Jul 13;16(9):jkag188. doi: 10.1093/g3journal/jkag188 (PMC13535368; doi:10.1093/g3journal/jkag188)
Supplement: Supplementary file 1 [file jkag188_supplementary_data.zip › Table_S1_G3-2026-407030.pdf]

Table S1: Number of chromosomes per species as reported by the literature and the number of contigs present in the assembly. Species that had chromosome number within 1 of the number of contigs were chosen for additional analysis.

Species	Literature chromosomes	Literature source	Number of contigs
Saprochaete fungicola	5	https://journals.asm.org/doi/10.1128/mra.00092-19	6
Candida parapsilosis	8	https://pmc.ncbi.nlm.nih.gov/articles/PMC6431126/	8
Clavispora lusitaniae	8	https://pmc.ncbi.nlm.nih.gov/articles/PMC6935856/#:~:text=Five%20independent%20assemblies%20were%20obtained,and%20P4%20(see%20below).	9
Meyerozyma guilliermondii	8	https://pmc.ncbi.nlm.nih.gov/articles/PMC12720209/#:~:text=Data%20summary,1).	9
Ashbya aceri	7	https://academic.oup.com/g3journal/article/3/8/1225/6025766	8
Eremothecium gossypii	7	https://www.ncbi.nlm.nih.gov/datasets/genome/GCA_000091025.4/	7
Eremothecium sinecaudum	8	https://www.ncbi.nlm.nih.gov/datasets/genome/GCF_001548555.1/#:~:text=Table_title:%20Assembly%20statistics%20Table_content:%20header:%20%7C%20%7C,%7C%20RefSeq:%207%20%7C%20GenBank:%207%20%7C	8
Yarrowia lipolytica	6	https://www.mdpi.com/2309-608X/7/7/548	6
Kluyveromyces lactis	6	https://www.ncbi.nlm.nih.gov/datasets/genome/GCA_000002515.1/#:~:text=Assembly%20methods,.labri.fr/Genolevures.	6
Saccharomyces cerevisiae	16	https://pmc.ncbi.nlm.nih.gov/articles/PMC3962479/#:~:text=Research%20regarding%20the%20genetics%20of,Oliver%2C%20England	16
Kazachstania naganishii	12	https://www.nature.com/articles/s42003-023-05285-0	13
Saprochaete ingens	5	https://pmc.ncbi.nlm.nih.gov/articles/PMC6908801/	6
Candida dubliniensis	8	https://www.kegg.jp/kegg-bin/show_organism?org=cdu	8
Scheffersomyces stipitis	8	https://www.kegg.jp/kegg-bin/show_organism?org=pic	9
Candida albicans	8	https://journals.asm.org/doi/10.1128/mbio.01205-18	9
Blastobotrys adeninivorans	4	https://pmc.ncbi.nlm.nih.gov/articles/PMC4022394/	4

Saccharomyces paradoxus	16	https://www.ncbi.nlm.nih.gov/datasets/genome/GCF_002079055.1/	16
Komagataella phaffii	4	https://www.ncbi.nlm.nih.gov/datasets/genome/GCA_900235035.2/#:~:text=Download%20datasets%20API%20FTP,phaffii%20(Pichia%20pastoris)%20CBS7435.	4
Ogataea parapolymorpha	7	https://www.ncbi.nlm.nih.gov/datasets/genome/GCF_000187245.1/	7
Candida orthopsilosis	8	https://pmc.ncbi.nlm.nih.gov/articles/PMC3338533/#:~:text=Our%20final%20C.,involving%20more%20than%20one%20gene.	8
Ogataea polymorpha	7	https://pmc.ncbi.nlm.nih.gov/articles/PMC9019687/#:~:text=The%20full%2Dlength%20genome%20of%20*Ogataea%20polymorpha*%20HU%2D11/CBS4732,structural%20variations%20between%20HU%2D11/CBS4732%20and%20NCYC495%20genomes	7
Saccharomyces kudriavzevii	16	https://www.ncbi.nlm.nih.gov/datasets/genome/GCF_947243775.1/#:~:text=Table_title:%20Assembly%20statistics%20Table_content:%20header:%20%7C%20%7C,%7C%20RefSeq:%2016%20%7C%20GenBank:%2016%20%7C	16
Saccharomyces mikatae	16	https://www.ncbi.nlm.nih.gov/datasets/genome/GCF_947241705.1/	16
Lachancea cidri	8	https://journals.asm.org/doi/10.1128/msystems.01058-23#:~:text=Here%2C%20we%20used%20a%20combination,in%20the%20strain's%20phenotypic%20variation.	8
Lachancea fantastica nom. nud.	7 or 8	https://journals.plos.org/plosone/article?id=10.1371/journal.pone.0047834 or https://forge.inrae.fr/gryc-data/lachancea_fantastica-cbs_6924/-/tree/master?ref_type=heads	7
Eremothecium cymbalariae	8	https://pmc.ncbi.nlm.nih.gov/articles/PMC3276169/	8
