# Supplementary material for: Genes near tRNAs are enriched in translational machinery
Source: G3 (Bethesda). 2026 Jul 13;16(9):jkag188. doi: 10.1093/g3journal/jkag188 (PMC13535368; doi:10.1093/g3journal/jkag188)
Supplement: Supplementary file 1 [file jkag188_supplementary_data.zip › Table_S2_G3-2026-407030.pdf]

Table S2: The different groups distance from tRNA was tested for normality with the Shapiro Wilks and Anderson Darling tests. These tests reveal that the data is not normally distributed, and is in agreement with the non-normality results from the qqplots. Therefore a Wilcoxin rank-sum test will be used for statistical testing.

Group	n	shapiro_p	ad_p	shapiro_normal	ad_normal
**Aminoacyl_tRNA**	2303	5.49498527327931E-07	3.7E-24	No	No
**Fatty_Acid**	395	5.53315769272589E-13	8.2400835445722E-21	No	No
**Glycolysis**	713	9.67753981224753E-13	2.06627507867344E-23	No	No
**Proteasome**	1930	1.10759053981797E-09	3.7E-24	No	No
**Ribosomal_Proteins**	6262	1.36239446986738E-10	3.7E-24	No	No
**Ribosome_Pathway**	4636	3.49617191642406E-10	3.7E-24	No	No
**rRNA_Infernal**	3799	4.761939513843E-13	3.7E-24	No	No
**tRNA_Modification**	3667	5.30593275913337E-09	3.7E-24	No	No
