# Supplementary material for: Genes near tRNAs are enriched in translational machinery
Source: G3 (Bethesda). 2026 Jul 13;16(9):jkag188. doi: 10.1093/g3journal/jkag188 (PMC13535368; doi:10.1093/g3journal/jkag188)
Supplement: Supplementary file 1 [file jkag188_supplementary_data.zip › Table_S3_G3-2026-407030.pdf]

Table S3: KEGGs related to neurological function were enriched near tRNAs. Since yeast do not have a neurological system, the KEGG IDs were further investigated and grouped into plausible pathways. This table explains the grouping methodology.

<table>
<tr><th>Group</th><th>Total_Count</th><th>Num_KEGGs</th><th>REGEX</th></tr>
<tr><td>Ion Transport</td><td>320</td><td>55</td><td>transporter|channel|P-type.*|VDAC|voltage-dependent anion channel|solute carrier|NADH dehydrogenase|F-type H\\+-transporting ATPase|succinate dehydrogenase</td></tr>
<tr><td>Proteasome</td><td>217</td><td>31</td><td>Proteasome</td></tr>
<tr><td>Mitochondrial / Redox</td><td>16</td><td>7</td><td>cytochrome|ubiquinol|oxidase|superoxide|catalase|glutathione|mitofusin|OMA1</td></tr>
<tr><td>Signaling / Kinases</td><td>39</td><td>11</td><td>kinase|MAPK|mTOR|phosphatase</td></tr>
<tr><td>Transcription / Nuclear transport</td><td>33</td><td>32</td><td>RNA polymerase|transcription|TFIID|histone|nuclear|nucleoporin|export</td></tr>
<tr><td>Autophagy</td><td>30</td><td>6</td><td>autophagy|beclin|ULK|ATG</td></tr>
<tr><td>Cytoskeleton</td><td>21</td><td>4</td><td>Tubulin|kinesin|dynactin</td></tr>
<tr><td>Ubiquitin system</td><td>20</td><td>9</td><td>ubiquitin|E1|E2|NEDD8</td></tr>
<tr><td>Other</td><td>15</td><td>13</td><td></td></tr>
<tr><td>Other signaling / Regulatory</td><td>11</td><td>5</td><td>Ras|calmodulin|thioredoxin</td></tr>
<tr><td>Chaperones / ER</td><td>9</td><td>5</td><td>BiP|stress-induced|peptidyl-prolyl|heat shock|ATP-dependent RNA/DNA helicase</td></tr>
<tr><td>Vesicle / Transport / Trafficking</td><td>7</td><td>6</td><td>clathrin|AP-2|TOM40|SEC13</td></tr>
<tr><td>Translation / Ribosome</td><td>5</td><td>1</td><td>translation initiation factor|ribosomal|ubiquitin-large subunit ribosomal</td></tr>
<tr><td>Total</td><td>743</td><td>185</td><td></td></tr>
</table>
